# Commentary: Thoracoscopic ablation for the faint of heart

**DOI:** 10.1016/j.xjtc.2021.04.027

**Published:** 2021-04-27

**Authors:** Donald D. Glower

**Affiliations:** Duke University Medical Center, Durham, NC

**Figure:**
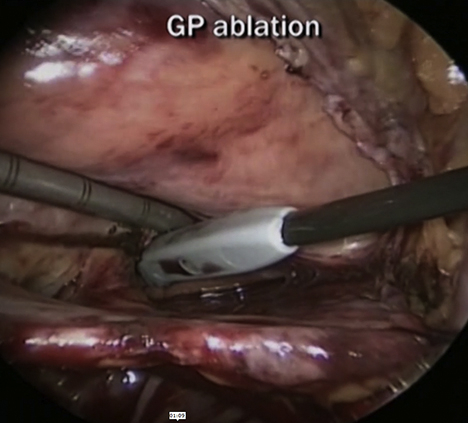
Bilateral thoracoscopic atrial ablation in patients with reduced ejection fraction.

**Central Message**

Bilateral thoracoscopic atrial ablation in patients with reduced ejection fraction can improve ventricular function and restore sinus rhythm.

See Article page 60.

**Figure 1:**
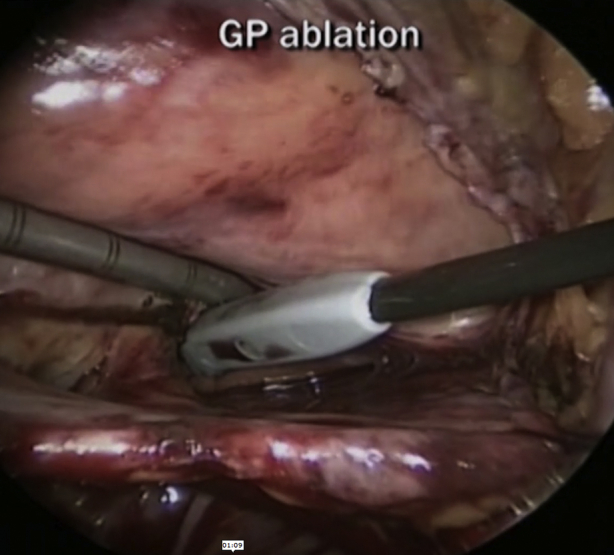
Bilateral thoracoscopic atrial ablation in patients with reduced ejection fraction.

Kim and colleagues^1^ present a series of 31 patients with reduced ejection fraction and persistent atrial fibrillation (AF) undergoing bilateral total thoracoscopic atrial ablation (TTA) (Figure 1). The authors concluded that TTA is a safe and effective procedure that improves left ventricular function and restores sinus rhythm in patients with AF and left ventricular dysfunction.

This is neither the first report of TTA,^2^ nor is it the first report of surgical AF ablation in patients with reduced ejection fraction.^3^ However, this may be one of the first series of TTA in patients with reduced ejection fraction. While the series is small, the follow-up is good. The illustrative video is helpful. These data reinforce the few studies of TTA and the few studies of surgical ablation in reduced ejection fraction.

This study does have limitations. First, this is a retrospective clinical series with echocardiography and rhythm assessment at irregularly timed intervals. Also of note, 8 of 31 patients did receive additional catheter ablation after the initial TTA procedure. Results are presented for all 31 patients after the last performed procedure, emphasizing the value of combined transcatheter and thoracoscopic approaches in some patients.

Thoracoscopy is not likely to replace catheter ablation, given the additional morbidity of surgical TTA over a transcatheter procedure. However, these data and others suggest that TTA may be a reasonable alternative to transcatheter ablation in patients who are low risk or who have failed transcatheter ablation or who may have other risk factors for transcatheter ablation failure, such as persistent AF. Thoracoscopic and other epicardial approaches do have advantages of access to some of the epicardial ganglion plexuses, which may be important in certain patients. Epicardial approaches can also provide access to externally occlude the left atrial appendage.^4^ In short, combined thoracoscopic and transcatheter approaches may be complementary. Other less-invasive surgical approaches such as the transpericardial convergent procedure^5^ may share with TTA the advantages and disadvantages of an epicardial approach over transcatheter endocardial procedures.

Thus, Kim and colleagues show that TTA can be safe and effective in patients with reduced ejection fraction. Thoracoscopic ablation may have a role, even for the faint of heart.
